# The Use of the Mullein Plant in the Treatment of Pulmonary Consumption

**Published:** 1883-03

**Authors:** 


THE USE OF THE MULLEIN PLANT IN THE TREATMENT OF PULMONARY CONSUMPTION.

F. J. B. Quinlan, M.D., M.R.I.A., F.K.Q.C.P., Physician at St. Vincent's Hospital, Dublin, observes that "from time immemorial the verbascum thapsus, or great mullein has been a trusted popular remedy, in Ireland, for the treatment of phthisis." After relating seven cases where it proved of benefit, he concludes, "I have set down the above cases simply in the order in which they occurred, and with no view of supporting any preconceived idea. These cases, although too few to justify any general conclusion, appear to establish some useful facts. The mullein plant boiled in milk is liked by the patients; in watery infusion it is disagreeable, and the succus is still more so. The hot milk decoction causes a comfortable (what the Gallic neighbors call *pectorale*) sensation, and when once patients take it they experience a physiological want, and when the supply was once or twice interrupted, complained much in consequence. That it eases phthisical cough, there can be no doubt; in fact, some of the patients scarcely took their cough mixtures at all—an unmixed boon to phthisical sufferers with delicate stomachs. Its power of checking phthisical looseness of the bowels was very marked, and experiment proved that this was not merely due to the well known astringent properties of boiled milk. It also gave great relief to the dyspnœa. For phthisical night-sweats it is utterly useless; but these can be completely checked by the hyperdermic use of, from oneeightieth to one-fiftieth of a grain of the atropia sulphate; the smaller dose, if it will answer, being preferable, as the larger causes dryness of the pharynx, and interferes with ocular accomodation. In advanced cases, it does not prevent loss of weight, nor am I aware of anything that will, except koumiss. Dr. Carrick, in his interesting work on the koumiss treatment of Southern Russia (page 213) says: 'I have seen a consumptive invalid gain largely in weight, while the disease was making rapid progress in her lungs, and the evening temperature rarely fell below 101° Fahr. Until then, I considered that an increase of weight in phthisis pulmonalis was a proof of the arrest of the malady.' If koumiss possessed this power, mullein clearly does not; but unfortunately, as real koumiss can be made from the milk of the mare only, and as it does not bear traveling, the consumptive invalid must go at least to Samara or Southern Russia. In pretubercular and early cases of pulmonary consumption, mullein appears to have a distinct weight-increasing power; and I have observed this in several private cases also. Having no weighings of these latter, however, makes this statement merely an expression of opinion. In early cases, the mullein milk appears to act very much in the same manner as cod-liver oil; and when we consider that it is at once cheap and palatable, it is certainly worth a trial. I will continue the research by careful weighings of early cases; and will further endeavor to ascertain whether the addition of mullein to the cultivating solution prevents the propagation of the phthisical bacillus."—*British Medical Journal.*

